# Heart attack and stroke occurrence at the intersection of race and sexual orientation: a nationally representative study of adults in the United States

**DOI:** 10.1186/s12889-025-24444-y

**Published:** 2025-10-03

**Authors:** Phoebe Tran, Brittany Shelton, Jennifer M. Jabson Tree

**Affiliations:** 1 Department of Public Health, University of Tennessee, Knoxville, TN USA; 2 Department of Public Health, Purdue University, 812 Mitch Daniels Blvd West Lafayette, West Lafayette, IN USA

**Keywords:** Myocardial infarction, Stroke, Intersectionality, Sexual orientation, Race

## Abstract

**Background:**

Strokes and myocardial infarctions (MIs) are among the most costly and quality of life diminishing conditions. Marginalized and minoritized people including Black, Indigenous, and other People of Color, experience higher MI and stroke odds than White comparisons. This may also be true for lesbian, gay, bisexual people (LGB) who have higher levels of MI and stroke risk factors than heterosexual comparisons. However, extremely limited evidence describes either MI/stroke occurrence among US adults with these intersecting identities.

**Methods:**

Self-reported race/ethnicity, sexual orientation, and MI and stroke diagnoses were obtained from the 2019–2021 Behavioral Risk Factor Surveillance System. Interaction analyses within survey-weighted age-adjusted logistic regression modeling were used to calculate the relative excess risk due to interaction (RERI).

**Results:**

Being female of another race (not White or Black) and LGB had an additive association with MI occurrence; the combined influences of being female, another race, and LGB on MI occurrence was greater than the sum of the influences of being White and heterosexual. The RERI indicated that female adults who were of another race and LGB experienced a relative excess risk of MI occurrence 2-fold higher (RERI: 1.99, 95% Confidence Interval: 0.33–3.65, *p* = 0.04) as that of those who were White only or heterosexual only.

**Conclusions:**

MI occurrence is high for all LGB females, we found that MI occurrence is especially high for females with LGB and “another” racial/ethnic identities.

**Supplementary information:**

The online version contains supplementary material available at 10.1186/s12889-025-24444-y.

## Introduction

Cardiovascular diseases (CVD), account for more deaths than all cancers and chronic lower respiratory illnesses combined [1] and include myocardial infarction (MI) (also known as heart attacks) and stroke. In the United States, approximately 805,000 MIs [2] and 795,000 strokes [3] occur annually contributing to the estimated $363 billion in medical expenses and lost productivity attributed to heart disease and stroke [4]. 

Certain Black, Indigenous, and other people of color (BIPoC) groups experience higher levels of modifiable MI and stroke risk factors and greater mortality for these two conditions than White comparisons. Estimates from nationally representative surveys show that some BIPoC groups had a higher prevalence of smoking (non-Hispanic American Indian/Alaska Native: 22.6%, non-Hispanic White: 15.0%) [5], hypertension (Black: 45.3%, White: 31.4%) [6], and physical inactivity (Hispanic: 31.7%, non-Hispanic Black: 30.3%, non-Hispanic White: 23.4%) [7] than White counterparts. The MI mortality rate among non-Hispanic Blacks was 46.7% higher than that of non-Hispanic Whites [8]. Non-Hispanic Blacks experienced a stroke prevalence and stroke mortality rate that were respectively 48.1% and 47.2% higher than that of non-Hispanic Whites [9]. 

Lesbian, gay, and bisexual (LGB) people are another group that appear to experience greater CVD burden. In their systematic review, Caceres and colleagues reported greater odds of having multiple MI and stroke risk factors among bisexual cisgender men compared to heterosexual cisgender men [10]. LGB identified populations have higher rates of smoking, and lesbian cisgender populations have higher rates of obesity [11] which are both MI and stroke risk factors. Additionally, control of CVD risk factors in this population may be suboptimal with a study surveying LGB individuals on statin use to prevent MI and stroke finding that LGB individuals were 63% less likely to use statins than individuals who did not identify as LGB [12]. 

Despite documentation concerning CVD burden in the form of MI and stroke occurrence in BIPoC, BIPoC are not a monolith. They hold multiple, intersecting, marginalized social identities, including LGB identities. MI and stroke health disparities are well documented separately among BIPoC and LGB populations, but much less is empirically known about odds for MI and stroke among BIPoC individuals with diverse sexual orientations. Further, the existing empirical evidence concerning MI and stroke among BIPoC and LGB groups predominantly applies reductionist frameworks and quantitative techniques that assume that social identities and categories are discrete [13]. This is problematic because it overly simplifies identities and the social experiences and contexts that may be risk factors for MI and stroke.

This investigation was guided by the intersectionality framework. Intersectionality was introduced by [14] and advanced by Black feminist scholars [14, 15], that describes how people who hold multiple intersecting marginalized identities are socially positioned within a multiplicity of social forces that shape one’s experiences and exposures to risk factors. Intersectionality is useful for conceptualizing how groups with multiple, intersecting, marginalized identities may be at greater odds for MI and stroke.

As such, our study sought to [1] document odds of ever having a MI and stroke among BIPoC individuals who are also LGB and [2] examine the interaction attributable to the intersection of race/ethnicity and LGB identity relative to race/ethnicity and sexual orientation independently. We hypothesized that LGB identity among BIPoC individuals will be associated with higher odds for ever having MI and stroke.

## Methods

### Study data and variables

Data were from the 2019, 2020, and 2021 nationally representative Behavioral Risk Factor Surveillance System (BRFSS) surveys. The BRFSS incorporates survey weighting to ensure that the sample of US adults participating in the BRFSS surveys is representative of the general non-institutionalized adult US population [16]. The BRFSS surveys, survey codebooks, and additional information on BRFSS survey methodology are available at the Centers for Disease Control and Prevention’s website [17]. 

We obtained information on MI and stroke status respectively through the BRFSS questions “(Ever told) you had a MI, also called a myocardial infarction?” and “(Ever told) (you had) a stroke.” [18–20] Individuals with “Don’t know/Not sure”, “Refused”, or “Not asked or Missing” responses to these questions were excluded from the study [18–20]. Sexual orientation was measured using a combination of the sex of respondent question and the separate sexual orientation questions for males and females. With these questions, males whose sexual orientation response was “Gay” or “Bisexual” were categorized as a LGB; “Straight, that is, not gay” as straight; and “Something else”, “I don ´t know the answer”, “Refused”, and “Not asked or Missing” were excluded [18–20]. Sexual orientation categories for females were determined the same way.

Race/ethnicity data came from the BRFSS’s imputed race values of “White, Non-Hispanic”, “Black, Non-Hispanic”, “Asian, Non-Hispanic”, “American Indian/Alaskan Native, Non-Hispanic”, “Hispanic”, and “Other race, Non-Hispanic” [18–20]. Due to the sparse responses in some of these race/ethnicity categories, we kept the “White, Non-Hispanic” and “Black, Non-Hispanic” groups as is but collapsed “Other race, Non-Hispanic”, “Asian, Non-Hispanic”, “American Indian/Alaskan Native, Non-Hispanic”, and “Hispanic” into “Other Race”. Unlike for MI, stroke, and sexual orientation, individuals with missing sociodemographic characteristics information were not excluded from the study. Hot-deck imputation was used to account for BRFSS survey weighting to fill in missing sociodemographic characteristics values [21]. 

### Statistical analyses

**Fig. 1:**
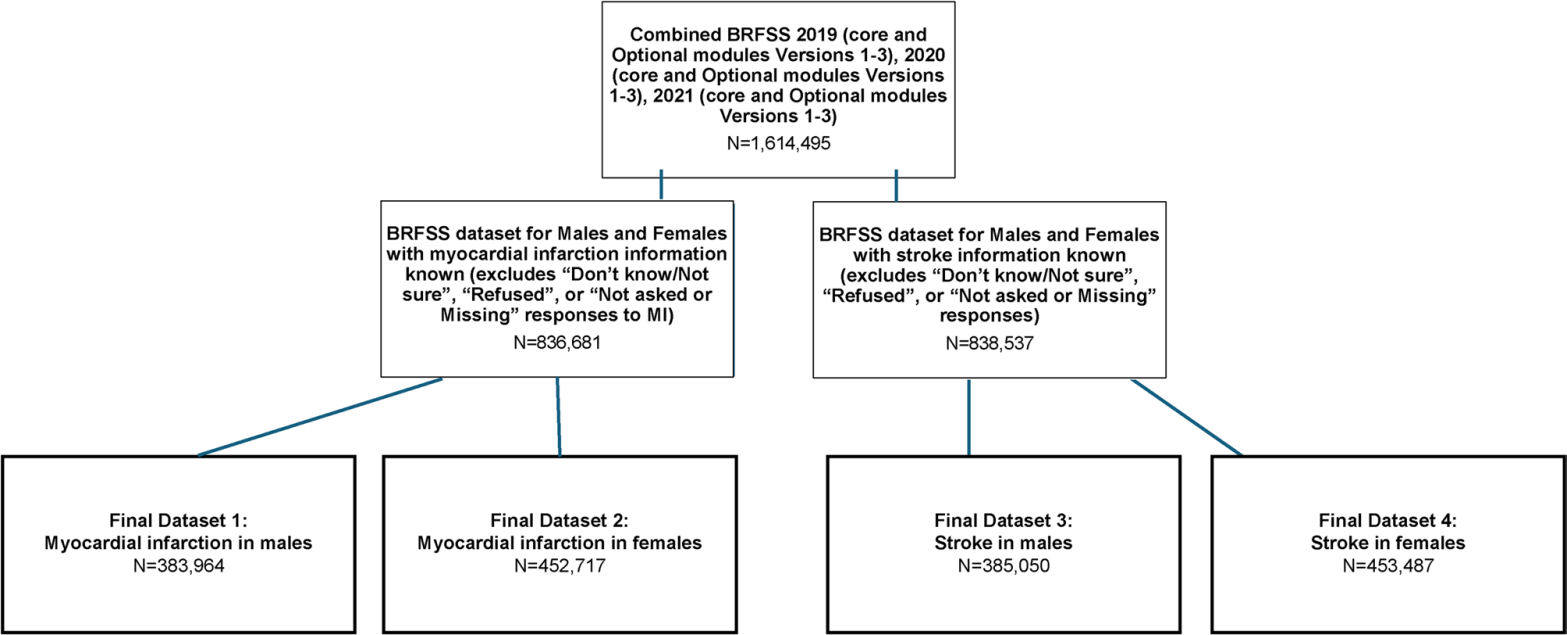
Flow diagram of data sets and participant inclusion

Figure 1 summarizes four separate datasets created to allow for the intersectional examination of the impact of sexual orientation and race/ethnicity on MI and stroke prevalence: [1] MI in males (*N* = 383,964), MI in females (*N* = 452,717) [3], stroke in males (*N* = 385,050), and [4] stroke in females (*N* = 453,487).

Within all of these datasets, we determined the distribution of the sociodemographic characteristics and the prevalence of either MI or stroke. Sex-specific logistic regression analyses, with sexual orientation and race/ethnicity as covariates, were calculated to assess the association of sexual orientation and race/ethnicity with either MI or stroke (Table S1). Age-adjusted estimates (18–44 years, 45–64 years, 65 + years) of the associations between sexual orientation, race/ethnicity, and either MI or stroke were also determined in this manner (Table S3). The sparse numbers of individuals in some sexual orientation and race/ethnicity strata limited our ability to adjust for additional sociodemographic characteristics.

We used the age-adjusted logistic regression model results to calculate the relative excess risk due to interaction (RERI). Models were not adjusted for clinical comorbidities. The RERI is a measure of additive interaction that represents the additional risk attributable to the interaction of two characteristics beyond what would be expected if they acted independently [22]. The existence of multiplicative interaction between sexual orientation and race/ethnicity in the age-adjusted logistic regression model was assessed by determining if the coefficient of the interaction term between these two factors was significantly different from zero.

Due to age being a known strong MI and stroke risk factor, we also examined two-way interactions between age and race and age and LGB identity as part of secondary analyses. Secondary analyses results are reported in TableS3. All analyses were survey-weighted and performed in SAS Version 9.4. Survey-weighted procedures conducted in SAS included PROC SURVEYFREQ and PROC SURVEYLOGISTIC. Statistical tests were two-sided and conducted at α = 0.05.

No human subjects were involved in this secondary analyses, therefore, IRB approval was not required.

## Results

Demographic characteristics of the sample are summarized in Table 1. Among males 4.7% identified as LGB and among females, 6.5% identified as LGB. Most males (66.8%) self-identified as White, non-Hispanic, 11.9% as African American/Black (heretofore referred to as Black), and 21.3% as Other Race, which included Asian, Pacific Islander, and Middle Eastern races. Similarly, most females identified as White (66.8%), 13.4% as Black, and 19.9% as Other Race.

**Table 1:** Distribution of sociodemographic characteristics and the prevalence of CVD conditions among 2019–2021 BRFSS survey respondents

	Myocardial infarction in males (*N* = 383,964)		Myocardial infarction in females (*N* = 452,717)		Stroke in males (*N* = 385,050)		Stroke in females (*N* = 453,487)	
	Frequency (*n*)	Weighted %	Frequency (*n*)	Weighted %	Frequency (*n*)	Weighted %	Frequency (*n*)	Weighted %
		(95% Confidence Interval)		(95% Confidence Interval)		(95% Confidence Interval)		(95% Confidence Interval)
Sexual Orientation								
Lesbian, Gay, Bisexual	15,271	4.7 (4.4, 4.9)	20,575	6.5 (6.2, 6.7)	15,286	4.6 (4.4, 4.9)	20,588	6.5 (6.2, 6.7)
Straight	368,693	95.3 (95.1, 95.6)	432,142	93.5 (93.3, 93.8)	369,764	95.4 (95.1, 95.6)	432,899	93.5 (93.3, 93.8)
Race/Ethnicity								
White, Non-Hispanic	300,697	66.8 (66.3, 67.3)	353,575	66.8 (66.3, 67.2)	301,294	66.7 (66.2, 67.2)	353,947	66.7 (66.3, 67.2)
Black, Non-Hispanic	24,399	11.9 (11.6, 12.3)	37,370	13.4 (13, 13.7)	24,583	12.0 (11.6, 12.4)	37,492	13.4 (13.0, 13.7)
Another Race	58,868	21.3 (20.8, 21.7)	61,772	19.9 (19.4, 20.3)	59,173	21.3 (20.9, 21.8)	62,048	19.9 (19.5, 20.3)
Age								
18-44y	117,708	46.1 (45.6, 46.6)	117,451	42.4 (41.9, 42.8)	118,007	46.1 (45.6, 46.6)	117,568	42.3 (41.8, 42.8)
45-64y	136,226	33.4 (32.9, 33.9)	159,522	33.5 (33.1, 33.9)	136,634	33.4 (33.0, 33.9)	159,775	33.5 (33.1, 33.9)
65 + y	130,030	20.5 (20.2, 20.8)	175,744	24.2 (23.8, 24.5)	130,409	20.5 (20.2, 20.8)	176,144	24.2 (23.9, 24.5)
Education								
Did not complete high school	23,587	11.5 (11.1, 11.9)	24,843	9.6 (9.3, 10.0)	23,825	11.6 (11.2, 12.0)	25,014	9.7 (9.3, 10.0)
High school graduate	104,116	29.8 (29.3, 30.2)	113,168	26.5 (26.1, 27)	104,581	29.8 (29.3, 30.2)	113,508	26.5 (26.1, 27.0)
Some College	102,074	29.5 (29.1, 30.0)	133,594	32.5 (32.0, 32.9)	102,279	29.5 (29.0, 30)	133,740	32.5 (32.0, 32.9)
College graduate	152,931	28.8 (28.4, 29.2)	179,995	31 (30.6, 31.4)	153,085	28.7 (28.3, 29.1)	180,086	31 (30.6, 31.4)
Missing	1256	0.4 (0.3, 0.5)	1117	0.3 (0.3, 0.4)	1280	0.4 (0.3, 0.5)	1139	0.3 (0.3, 0.4)
Income								
Less than $15,000	19,296	5.1 (4.9, 5.4)	30,731	6.9 (6.6, 7.1)	19,428	5.2 (5.0, 5.4)	30,857	6.9 (6.7, 7.1)
$15,000 to less than $25,000	35,639	9.8 (9.5, 10.1)	55,454	12.3 (12.0, 12.6)	35,883	9.8 (9.5, 10.2)	55,603	12.3 (12.0, 12.7)
$25,000 to less than $35,000	31,402	8.0 (7.7, 8.3)	42,414	8.9 (8.7, 9.2)	31,466	8.0 (7.7, 8.3)	42,548	9.0 (8.7, 9.2)
$35,000 to less than $50,000	44,066	10.5 (10.3, 10.8)	51,111	10.3 (10.1, 10.6)	44,207	10.5 (10.2, 10.8)	51,144	10.3 (10.0, 10.6)
$50,000 or more	195,076	49.0 (48.5, 49.5)	184,686	40.4 (39.9, 40.8)	195,270	48.9 (48.4, 49.4)	184,750	40.3 (39.9, 40.8)
Missing	58,485	17.5 (17.1, 17.9)	88,321	21.1 (20.7, 21.5)	58,796	17.5 (17.1, 17.9)	88,585	21.2 (20.8, 21.6)
Health Insurance								
Has insurance	229,121	58.1 (57.7, 58.5)	283,219	60.7 (60.4, 61.1)	229,653	58.1 (57.7, 58.5)	283,682	60.7 (60.4, 61.1)
No insurance	23,324	9.0 (8.7, 9.4)	20,283	6.7 (6.4, 7.0)	23,499	9.1 (8.7, 9.4)	20,363	6.7 (6.4, 7.0)
Missing	131,519	32.8 (32.6, 33.1)	149,215	32.6 (32.3, 32.8)	131,898	32.8 (32.6, 33.1)	149,442	32.6 (32.3, 32.8)
Heart Attack								
Had a heart attack	28,611	5.5 (5.3, 5.7)	18,373	3.2 (3.0, 3.3)	--	--	--	--
No heart attack	355,353	94.5 (94.3, 94.7)	434,344	96.8 (96.7, 97)	--	--	--	--
Stroke								
Had a stroke	--	--	--	--	16,376	3.4 (3.2, 3.5)	19,726	3.6 (3.4, 3.7)
No stroke	--	--	--	--	368,674	96.6 (96.5, 96.8)	433,761	96.4 (96.3, 96.6)

MI and stroke prevalence by sexual orientation and race/ethnicity, stratified by sex assigned at birth, is summarized by Tables 2 and 3.

**Table 2:** Prevalence of CVD conditions by sex, sexual orientation, and race/ethnicity. Myocardial infarction by sex, sexual orientation, and race/ethnicity

Group	MI Status	Frequency	Weighted % (95% CI)
Males			
White, Non-Hispanic (Lesbian, Gay, Bisexual)	Had a heart attack	728	3.5 (2.9, 4.1)
	No heart attack	10,536	96.5 (95.9, 97.1)
White, Non-Hispanic (Straight)	Had a heart attack	23,219	6.2 (6.0, 6.4)
	No heart attack	266,214	93.8 (93.6, 94.0)
Black, Non-Hispanic (Lesbian, Gay, Bisexual)	Had a heart attack	43	2.5 (0.9, 5.0)
	No heart attack	934	97.6 (96.0, 99.1)
Black, Non-Hispanic (Straight)	Had a heart attack	1382	4.8 (4.0, 5.5)
	No heart attack	22,040	95.3 (94.5, 96.0)
Another Race (Lesbian, Gay, Bisexual)	Had a heart attack	132	3.8 (1.6, 6.0)
	No heart attack	2898	96.2 (94.0, 98.4)
Another Race (Straight)	Had a heart attack	3107	4.4 (3.8, 5.0)
	No heart attack	52,731	95.6 (95.0, 96.2)
Females			
White, Non-Hispanic (Lesbian, Gay, Bisexual)	Had a heart attack	348	1.6 (1.1, 2.1)
	No heart attack	14,440	98.4 (98.0, 98.9)
White, Non-Hispanic (Straight)	Had a heart attack	14,148	3.5 (3.3, 3.6)
	No heart attack	324,639	96.5 (96.4, 96.7)
Black, Non-Hispanic (Lesbian, Gay, Bisexual)	Had a heart attack	53	2.2 (0.5, 3.8)
	No heart attack	1487	97.9 (96.2, 99.5)
Black, Non-Hispanic (Straight)	Had a heart attack	1688	3.3 (2.9, 3.8)
	No heart attack	34,142	96.7 (96.2, 97.1)
Another Race (Lesbian, Gay, Bisexual)	Had a heart attack	137	4.3 (2.3, 6.4)
	No heart attack	4110	95.7 (93.6, 97.7)
Another Race (Straight)	Had a heart attack	1999	2.2 (1.9, 2.6)
	No heart attack	55,526	97.8 (97.4, 98.1)

**Table 3:** Stroke by sex, sexual orientation, and race/ethnicity

Group	Stroke Status	Frequency	Weighted % (95% CI)
Males			
White, Non-Hispanic (Lesbian, Gay, Bisexual)	Had a stroke	420	2.9 (1.9, 3.8)
	No stroke	10,851	97.1 (96.2, 98.1)
White, Non-Hispanic (Straight)	Had a stroke	12,443	3.4 (3.2, 3.6)
	No stroke	277,580	96.6 (96.4, 96.8)
Black, Non-Hispanic (Lesbian, Gay, Bisexual)	Had a stroke	62	3.0 (1.5, 4.6)
	No stroke	914	97.0 (95.4, 98.5)
Black, Non-Hispanic (Straight)	Had a stroke	1434	4.9 (4.2, 5.7)
	No stroke	22,173	95.1 (94.3, 95.8)
Another Race (Lesbian, Gay, Bisexual)	Had a stroke	83	2.9 (1.1, 4.8)
	No stroke	2956	97.1 (95.2, 98.9)
Other (Straight)	Had a stroke	1934	2.5 (2.2, 2.9)
	No stroke	54,200	97.5 (97.1, 97.9)
Females			
White, Non-Hispanic (Lesbian, Gay, Bisexual)	Had a stroke	433	1.9 (1.4, 2.3)
	No stroke	14,350	98.1 (97.7, 98.6)
White, Non-Hispanic (Straight)	Had a stroke	14,789	3.7 (3.6, 3.9)
	No stroke	324,375	96.3 (96.1, 96.4)
Black, Non-Hispanic (Lesbian, Gay, Bisexual)	Had a stroke	73	3.4 (1.4, 5.5)
	No stroke	1466	96.6 (94.5, 98.6)
Black, Non-Hispanic (Straight)	Had a stroke	2271	5.0 (4.4, 5.5)
	No stroke	33,682	95.0 (94.5, 95.6)
Another Race (Lesbian, Gay, Bisexual)	Had a stroke	121	3.5 (1.6, 5.4)
	No stroke	4145	96.5 (94.6, 98.4)
Another Race (Straight)	Had a stroke	2039	2.4 (1.9, 2.9)
	No stroke	55,743	97.6 (97.1, 98.1)

## Additive and multiplicative interactions for MI

### Males

Additive and multiplicative interactions for MI among males are summarized by Table 4. Compared to non-LGB White males, being LGB and a White male was not significantly associated with odds of MI (adjusted odds ratio (aOR): 0.98, 95% CI: 0.79–1.21), nor was being LGB and a Black male (aOR: 0.88, 95% CI: 0.54–1.43) (Table 4). There was no statistically significant association between Black race and odds of MI without the presence of LGB status (aOR: 0.95, 95% CI: 0.81–1.13). Among Black individuals, LGB identification was not significantly associated with odds of MI (aOR: 0.92, 95% CI: 0.65–1.30). Correspondingly, there was no significant interaction detected on either the multiplicative scale (aOR: 0.94, 95% CI: 0.80–1.11) or the additive scale (RERI: −0.05, 95% CI: −0.13-0.02, *p* = 0.17).

**Table 4:** Interaction analysis results. Myocardial infarction and interaction of sex, sexual orientation, and race/ethnicity

	Lesbian, Gay, Bisexual	Straight	Odds Within the specified racial/ethnic and sex group, comparing Lesbian, Gay, Bisexual to Straight
Individual Factors	aOR (95% CI) ^A^	aoR (95% CI) ^A^	aOR within strata ^A^
*Male*			
Race			
White	0.98 (0.79–1.21)	Ref	0.98 (0.79–1.21)
Black	0.88 (0.54–1.43)	0.95 (0.81–1.13)	0.92 (0.65–1.30)
LGB*Black	0.94 (0.80–1.11)		
RERI: −0.05 (−0.13, 0.02)			RERI *p* = 0.17
Race			
White	0.98 (0.79–1.21)	Ref	0.98 (0.79–1.21)
Another Race	1.31 (0.83–2.06)	1.22 (1.05–1.42)	1.07 (0.77–1.48)
LGB*Another Race	1.09 (0.94–1.27)		
RERI: 0.11 (−0.15, 0.36)			RERI *p* = 0.42
*Female*			
Race			
White	**1.38 (1.10–1.74)**	Ref	**1.38 (1.10–1.74)**
Black	1.72 (0.96–3.11)	1.17 (0.96–1.44)	1.47 (0.98–2.20)
LGB*Black	1.06 (0.87–1.30)		
RERI: 0.17 (−0.37, 0.65)			RERI *p* = 0.49
Race			
White	**1.38 (1.10–1.74)**	Ref	**1.38 (1.10–1.74)**
Another Race	**2.50 (1.62–3.86)**	**1.43 (1.23–1.66)**	**1.75 (1.26–2.43)**
LGB*Another Race	**1.26 (1.09–1.47)**		
RERI:**1.99 (0.33**,** 3.65)**			RERI *p* = 0.04

^A^ Bold indicates significance at *p* < 0.05

Being LGB and a male of another race was not significantly associated with odds of MI (aOR: 1.31, 95% CI: 0.83–2.06) (Table 4), but identification as a non-LGB male of another race was associated with 22% higher odds of MI (aOR: 1.22, 95% CI: 1.05–1.42). Among males of another self-reported race, LGB identification was not associated with odds of MI (aOR: 1.07, 95% CI: 0.77–1.48). Neither the multiplicative scale (aOR: 1.09, 95% CI: 0.94–1.27) or the additive scale (RERI: 0.11, 95% CI: −0.15-0.36, *p* = 0.42) demonstrated any evidence of a significant interaction between another race and LGB-identification with MI.

### Females

In comparison to non-LGB White females, LGB White females had a 38% higher odds of MI (adjusted odds ratio (aOR): 1.38, 95% CI: 1.10–1.74) (Table 4). Being both LGB and a Black female was not associated with increased MI odds (aOR: 1.72, 95% CI: 0.96–3.11), nor was there a significant association between Black race and MI without the presence of LGB identity (aOR: 1.17, 95% CI: 1.05–1.42). Among Black females, being LGB was not associated with odds of MI (aOR: 1.47, 95% CI: 0.98–2.20), and there was no significant interaction on either the multiplicative scale (aOR: 1.06, 95% CI: 0.87–1.30) or on the additive scale (RERI: 0.17, 95% CI: −0.37-0.65, *p* = 0.49).

**Fig. 2:**
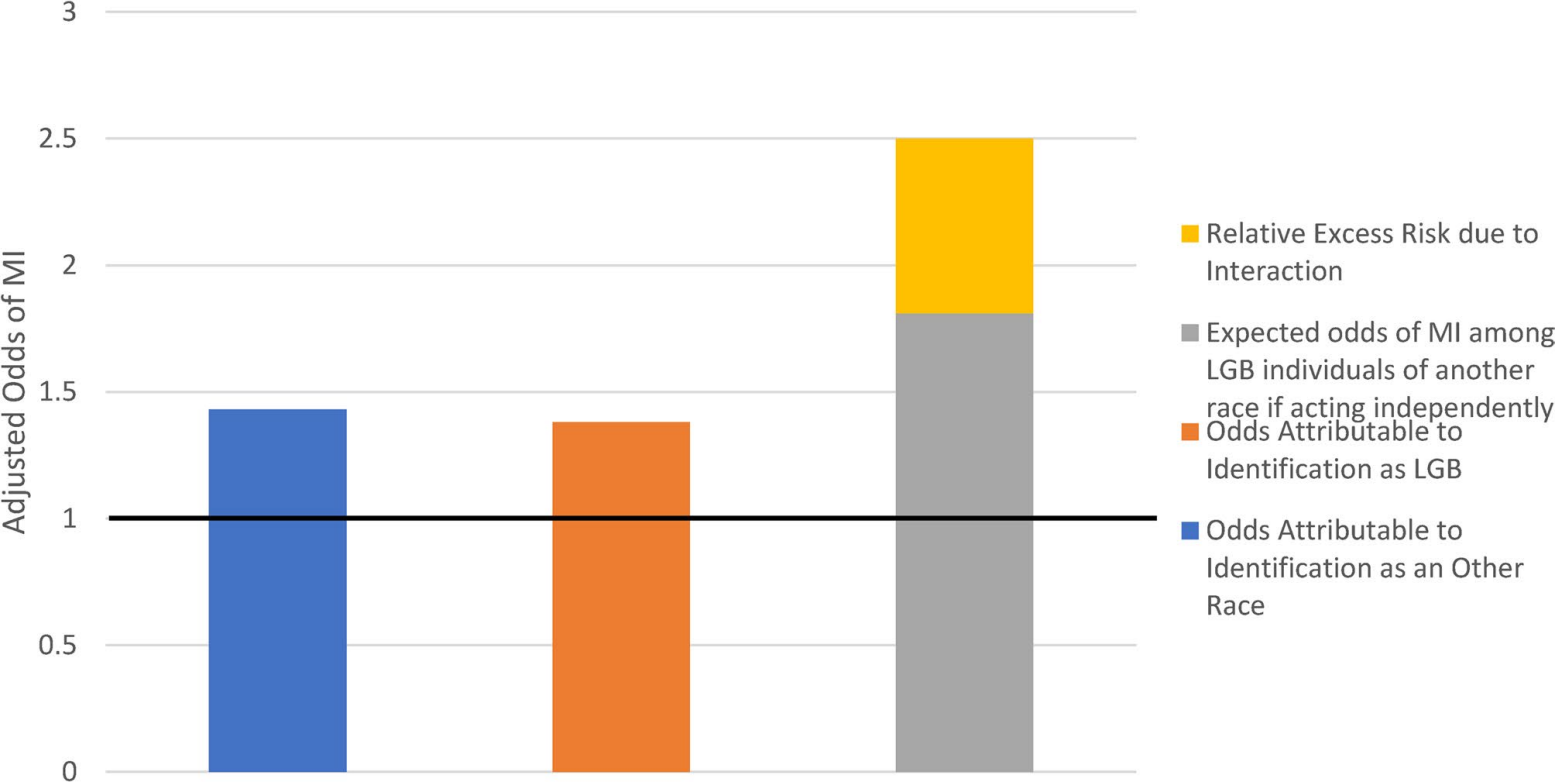
Relative Excess Risk of Myocardial Infarction among Females of “Other” Self-Identified Race ^i^The first column illustrates the odds of myocardial infarction associated with identifying as a heterosexual female of Another race. The second column illustrates the odds of myocardial infarction associated with self-identifying as lesbian, gay, bisexual White female. In the third column, the gray component illustrates what the expected odds of MI would be for a lesbian, gay, bisexual woman self-identifying as belonging to an another race if those odds acted additively. The yellow portion of the third bar illustrates the relative excess risk due to interaction or the amount of additional risk identified in this study that can be allocated to the interaction between race and sexual orientation among lesbian, gay, bisexual females identifying as belonging to an another race

Being LGB and female of ‘other’ race was associated with 2.5 times the odds of MI, compared to non-LGB, White, females (aOR: 2.50, 95% CI: 1.62–3.86) (Table 4). Females of ’other’ race had a 43% higher odds of MI without the presence of LGB status (aOR: 1.43, 95% CI: 1.23–1.66) and, among females of another race, LGB identity was associated with 75% higher odds of MI (aOR: 1.75, 95% CI: 1.26–2.43). Thus, there was a significant interaction on the multiplicative scale (aOR: 1.26, 95% CI: 1.09–1.47) and on the additive scale (RERI: 1.99, 95% CI: 0.33–3.65, *p* = 0.04). This means that being both LGB and a female of ‘another’ race was associated with increased odds of MI beyond what would be expected if being LGB and female of ‘another’ race acted independently (Fig. 2).

## Additive and multiplicative interactions for stroke

### Males

Compared to non-LGB White males, being LGB and White was not significantly associated with odds of stroke (adjusted odds ratio (aOR): 1.10, 95% CI: 0.88–1.37), nor was self-identification as both LGB and as Black race (aOR: 1.26, 95% CI: 0.79–2.03) (Table 5). Black race was significantly associated with 23% higher odds of stroke, compared to non-LGB White males (aOR: 1.23, 95% CI: 1.04–1.46) but, among Black males, LGB identification was not associated with odds of stroke (aOR: 1.02, 95% CI: 0.79–2.03). Thus, there was no significant interaction on the multiplicative scale (aOR: 0.93, 95% CI: 0.79–1.10) or on the additive scale (RERI: −0.07, 95% CI: −0.31-0.17, *p* = 0.55).

**Table 5:** Interaction analysis results. Stroke interaction of sex, sexual orientation, and race/ethnicity

	Lesbian, Gay, Bisexual	Straight	Odds Within the specified racial/ethnic and sex group, comparing Lesbian, Gay, Bisexual to Straight
Individual Factors	aOR (95% CI) ^A^	aoR (95% CI) ^A^	aOR within strata ^A^
*Male*			
Race			
White	1.10 (0.88–1.37)	Ref	1.10 (0.88–1.37)
Black	1.26 (0.79–2.03)	**1.23 (1.04–1.46)**	1.02 (0.79–2.03)
LGB*Black	0.93 (0.79–1.10)		
RERI: −0.07 (−0.31, 0.17)			RERI *p* = 0.55
Race			
White	1.10 (0.88–1.37)	Ref	1.10 (0.88–1.37)
Another Race	1.31 (0.76–2.25)	1.13 (0.93–1.37)	1.16 (0.79–1.69)
LGB*Another Race	1.05 (0.87–1.27)		
RERI: 0.07 (−0.23, 0.38)			RERI *p* = 0.64
Female			
Race			
White	**1.35 (1.09–1.69)**	Ref	**1.35 (1.09–1.69)**
Black	**2.04 (1.23–3.39)**	**1.40 (1.18–1.66)**	**1.46 (1.02–2.08)**
LGB*Black	1.08 (0.91–1.27)		
RERI: 0.29 (−0.28, 0.85)			RERI *p* = 0.32
Race			
White	**1.35 (1.09–1.69)**	Ref	**1.35 (1.09–1.69)**
Another Race	**2.07 (1.29–3.30)**	**1.23 (1.05–1.45)**	**1.68 (1.19–2.37)**
LGB*Another Race	**1.24 (1.05–1.46)**		
RERI: 0.48 (−0.08, 1.04)			RERI *p* = 0.09

*bold indicates significance at *p* < 0.05

Compared to non-LGB White males, being LGB and male of ’another’ race was not associated with odds of stroke (aOR: 1.31, 95% CI: 0.76–2.25), nor was being non-LGB male of ‘another’ race (aOR: 1.13, 95% CI: 0.93–1.37) (Table 5). Among males of ‘another’ race, being LGB was not associated with odds of stroke (aOR: 1.16, 95% CI: 0.79–1.69). Correspondingly, there was no significant interaction on the multiplicative scale (aOR: 1.05, 95% CI: 0.87–1.27) or on the additive scale (RERI: 0.07, 95% CI: −0.23-0.38, *p* = 0.64).

### Females

Compared to non-LGB White females, being LGB and White was associated with a 35% increased odds of stroke (aOR: 1.35, 95% CI: 1.09–1.69), and being both LGB and Black was associated with two times the odds of stroke (aOR: 2.04, 95% CI: 1.23–3.39) (Table 5). Black race was significantly associated with 40% higher odds of stroke without the presence of LGB identification (aOR: 1.40, 95% CI: 1.18–1.66) and, among Black females, being LGB was significantly associated with 46% higher odds of stroke (aOR: 1.46, 95% CI: 1.02–2.08). There was no significant interaction detected on either the multiplicative scale (aOR: 1.08, 95% CI: 0.91–1.27) or additive scale (RERI: 0.29, 95% CI: −0.28-0.85, *p* = 0.32).

Compared to non-LGB White females, being LGB and ‘another’ race was associated with significantly higher odds of stroke (aOR: 2.07, 95% CI: 1.29–3.30) as was being non-LGB female of ‘another’ race (aOR: 1.23, 95% CI: 1.05–1.45) (Table 5). Among females of ‘another’ race, LGB identification was significantly associated with higher odds of stroke (aOR: 1.68, 95% CI: 1.19–2.37). However, while there was a significant interaction present on the multiplicative scale (aOR: 1.24, 95% CI: 1.05–1.46), this interaction failed to meet statistical significance on the additive scale (RERI: 0.48, 95% CI: −0.08-1.04, *p* = 0.09).

## Discussion

BIPoC and LGB identified people are diverse groups made up of individuals with vibrant and diverse identities with many having multiple, marginalized, intersecting identities. Accordingly, we set out to investigate and document MI and stroke occurrence among BIPoC who are also LGB. We hypothesized that LGB identity among BIPoC individuals would be associated with increased CVD occurrence in the form of MI and stroke.

Our findings partially supported our hypotheses. We found that LGB females who identified as ‘other’ race experienced 26% greater odds of MI than LGB White females. Relative to race and sexual orientation identities independently, we found evidence that the interaction between sexual orientation and race was significant for females of another race. For females of another race, these intersecting identities were associated with two-fold higher odds of MI beyond what would be expected if sexual orientation and race acted independently. Our other hypotheses concerning odds for MI were not supported. Moreover, we did not find increased odds for MI among LGB BIPoC males. There was no evidence of additional influences of being LGB and BIPoC on stroke for either males or females.

Some, but not all, of our findings aligned with the extant published evidence. Caceres et al. [23] observed that LGB BIPOC males and females both experienced physiological risk factors for cardiovascular diseases. Similarly, we found that LGB females of color were at higher odds for MI and that the interaction effect between identities was especially significant in MI occurrence. However, unlike Caceres et al., [23], we did not find similar patterns in CVD occurrence among LGB BIPoC males.

Methodological differences, including our outcomes (self-reported MI) and our use of RERI to assess additive interaction, may explain why our findings differ from Caceres et al. [23] By examining the influence of sexual orientation and race on MI and stroke individually, we were able to identify a significant interaction between these factors for MI but not stroke. A potential reason for our observation may be that there are different pathophysiological pathways through which exposures contribute to MI and stroke. Another explanation could be that exposure to heterosexism and racism together lead to greater access barriers [24] to preventative care for MI than stroke. In their investigation of healthcare bias experienced by BIPoC LGBTQ + patients, Casanova-Perez and colleagues [25] found that patients who hold both identities as BIPoC and LGBTQ + experience power inequities, communication challenges, bias embedded in medical treatment as well as systemic issues within healthcare organizations. These included implicit discrimination exhibited toward BIPoC LGBTQ + patients, unfair treatment, and negative consequences experienced by BIPoC LGBTQ + patients after experiences of bias and discrimination. These experiences support the possible explanation that exposure to heterosexism and racism together negatively affect access to preventive care for MI.

There is much to learn about MI and stroke risk at the intersection of sexual orientation and race. Current evidence in this area is in its nascent stages. Although ours is among the first and growing number of epidemiological efforts to document joint disparity for MI and stroke risk among LGB females of color, it is not the first attempt at using these methods to understand the joint contributions of racism and heterosexism in health outcomes. In an intersectional analysis of health surveillance data sources, investigators [26] observed a protective effect in the context of food insecurity at the intersection of being Black and LGB among females. They suggested that it was possible that underlying cultural processes such as shared norms and cultural resilience could facilitate support networks that help minoritized groups cope with systemic oppression [27, 28]. 

### Limitations

The findings reported here have limitations. All BRFSS survey responses are self-reported. Nonetheless, there have been extensive validation studies conducted by the Centers for Disease Control and Prevention (CDC) and non-CDC affiliated investigators indicating that the BRFSS responses on medical history questions have high agreement with in-person measurements and electronic health records [29, 30]. 

The BRFSS does not measure structural or interpersonal racism and heterosexism. Consequently, we used race/ethnicity and sexual orientation as proxies to indicate exposure to racism and heterosexism. This is not aligned with best practices for intersectionality or anti-racist research [31–33]. Using sexual orientation and race/ethnicity as proxies for exposure to multi-level racism and heterosexism, may increase the false interpretation that disparities are caused by genetic factors, rather than social fundamental cause of disease [34]. 

Sexual orientation was assessed by asking participants to report on sexual identity, and excluded the other two dimensions of sexual orientation, attraction and behavior. Therefore, it is possible that some individuals who do not identify as LGB but due to sexual attraction and sexual behavior may experience heterosexism, homophobia, and marginalization, were not coded as such in our analyses. Additionally, due to the historic mistrust and marginalization perpetrated on LGB groups, it is possible that LGB people may be less likely to report their sexual orientation honestly in government sponsored health surveillance, resulting in under-reported LGB identities.

Due to small sample sizes we were forced to aggregate multiple identity groups including combining multiple racial and ethnic groups into a single “other race” group and combining all sexual orientations that were not heterosexual into one “LGB” group. This practice aligns with scientific norms concerning sample sizes and analytical practices, but it is in direct opposition to best practices in intersectional and anti-racism research. Future health surveillance efforts should be made to oversample groups including LGB adults and other racial/ethnic minoritized groups. Last, we excluded individuals who reported their sexual orientation as “Something else”. Those individuals could include those who identify as pansexual, asexual, demisexual, or hold some other sexual identity.

This study was also limited due to the cross-sectional design, making it impossible to determine temporality.

### Strengths

This project also had strengths including the use of health surveillance data that is meant to represent the US population. We also provided an in-depth analysis of the attributable risk of each marginalized identity to odds for MI and stroke.

## Conclusion

Using contemporary US data, we assessed the separate and joint influences of sexual orientation and race on MI and stroke occurrence. Our findings demonstrated that, while MI occurrence is high for all LGB females, MI occurrence is especially high for females with LGB and “another” racial/ethnic identities. Findings from this observational study provide foundational empirical evidence motivating additional clinical and qualitative research designed to understand and intervene on these inequities among BIPoC LGB individuals.

## Supplementary Information

Below is the link to the electronic supplementary material.

Supplementary Material 1

Supplementary Material 2

Supplementary Material 3

## Data Availability

Data are publicly available for free at https://www.cdc.gov/brfss/annual_data/annual_data.htm.

## Abbreviations

BIPoC: Black, Indigenous, and People of Color
LGB: Lesbian, gay, bisexual
LGBTQ+: Lesbian, gay, bisexual, transgender, queer +
MI: Myocardial infarction
CVD: Cardiovascular disease
US: United States
RERI: Relative Excess Risk due to Interaction
aOR: Adjusted odds ratio
SD: Standard deviation
