# Supplementary material for: Heart attack and stroke occurrence at the intersection of race and sexual orientation: a nationally representative study of adults in the United States
Source: BMC Public Health. 2025 Oct 3;25:3328. doi: 10.1186/s12889-025-24444-y (PMC12495760; doi:10.1186/s12889-025-24444-y)
Supplement: Supplementary file 1 — Supplementary Material 1 [file 12889_2025_24444_MOESM1_ESM.docx]

**Table S1.** Sex-specific logistic regression models with sexual orientation and race/ethnicity as covariates and with age adjustment.

| Not adjusted for age | | | | |
| --- | --- | --- | --- | --- |
|  | MI in males | MI in females | Stroke in males | Stroke in females |
|  | OR (95% CI) | OR (95% CI) | OR (95% CI) | OR (95% CI) |
| Race/ethnicity  (Reference: White) |  | | | |
| Black, Non-Hispanic | 0.75 (0.64, 0.89) | 0.97 (0.84, 1.12) | 1.45 (1.23, 1.72) | 1.36 (1.20, 1.54) |
| Other | 0.71 (0.62, 0.82) | 0.72 (0.61, 0.85) | 0.75 (0.64, 0.88) | 0.68 (0.56, 0.84) |
| Sexual minority status (Reference: Straight) |  | | | |
| Sexual minority | 0.60 (0.49, 0.75) | 0.75 (0.57, 0.99) | 0.86 (0.64, 1.14) | 0.69 (0.53, 0.89) |
| Adjusted for age | | | | |
|  | MI in males | MI in females | Stroke in males | Stroke in females |
|  | OR (95% CI) | OR (95% CI) | OR (95% CI) | OR (95% CI) |
| Race/ethnicity (Reference: White) |  |  |  |  |
| Black, Non-Hispanic | 0.91 (0.77, 1.08) | 1.24 (1.07, 1.43) | 1.75 (1.48, 2.07) | 1.71 (1.51, 1.94) |
| Other | 1.16 (1.01, 1.34) | 1.18 (0.99, 1.39) | 1.17 (0.99, 1.38) | 1.06 (0.85, 1.31) |
| Sexual minority status (Reference: Straight) |  | | | |
| Sexual minority | 0.93 (0.75, 1.16) | 1.62 (1.21, 2.17) | 1.28 (0.96, 1.72) | 1.38 (1.06, 1.79) |
| Age  (Reference: 65+y) |  | | | |
| 18-44y | 0.06 (0.05, 0.08) | 0.09 (0.07, 0.11) | 0.09 (0.07, 0.11) | 0.23 (0.20, 0.27) |
| 45-64y | 0.37 (0.34, 0.40) | 0.43 (0.39, 0.48) | 0.45 (0.41, 0.50) | 2.04 (1.83, 2.26) |
