# Supplementary material for: Heart attack and stroke occurrence at the intersection of race and sexual orientation: a nationally representative study of adults in the United States
Source: BMC Public Health. 2025 Oct 3;25:3328. doi: 10.1186/s12889-025-24444-y (PMC12495760; doi:10.1186/s12889-025-24444-y)
Supplement: Supplementary file 2 — Supplementary Material 2 [file 12889_2025_24444_MOESM2_ESM.docx]

**Table S2.** Assessment of significance of covariates in age-adjusted logistic regression models

**S2.1. Myocardial Infarction in Males**

| **Type 3 Analysis of Effects** | | | | |
| --- | --- | --- | --- | --- |
| **Effect** | **F Value** | **Num DF** | **Den DF** | **Pr > F** |
| **Race/ethnicity** | 2.85 | 2 | 210839 | 0.0579 |
| **Sexual orientation** | 0.44 | 1 | 210840 | 0.5076 |
| **age** | 663.28 | 2 | 210839 | <.0001 |

**S2.2. Myocardial infarction in females**

| **Type 3 Analysis of Effects** | | | | |
| --- | --- | --- | --- | --- |
| **Effect** | **F Value** | **Num DF** | **Den DF** | **Pr > F** |
| **Race/ethnicity** | 5.47 | 2 | 246386 | 0.0042 |
| **Sexual orientation** | 10.49 | 1 | 246387 | 0.0012 |
| **age** | 313.59 | 2 | 246386 | <.0001 |

**S2.3. Stroke in males**

| **Type 3 Analysis of Effects** | | | | |
| --- | --- | --- | --- | --- |
| **Effect** | **F Value** | **Num DF** | **Den DF** | **Pr > F** |
| **Race/ethnicity** | 21.88 | 2 | 211460 | <.0001 |
| **Sexual orientation** | 2.78 | 1 | 211461 | 0.0953 |
| **age** | 304.30 | 2 | 211460 | <.0001 |
