# Supplementary material for: Heart attack and stroke occurrence at the intersection of race and sexual orientation: a nationally representative study of adults in the United States
Source: BMC Public Health. 2025 Oct 3;25:3328. doi: 10.1186/s12889-025-24444-y (PMC12495760; doi:10.1186/s12889-025-24444-y)
Supplement: Supplementary file 3 — Supplementary Material 3 [file 12889_2025_24444_MOESM3_ESM.docx]

**Table S3.** Secondary analyses results of two-way interactions

**S3.1. Myocardial infarction in males**

| Analysis of Maximum Likelihood Estimates | | | | | | |
| --- | --- | --- | --- | --- | --- | --- |
| Parameter |  |  | Estimate | Standard Error | t Value | Pr > \|t\| |
| Intercept |  |  | -1.7129 | 0.0233 | -73.53 | <.0001 |
| race | Black, Non-Hispanic |  | -0.3461 | 0.1190 | -2.91 | 0.0036 |
| race | Other |  | -0.1062 | 0.1160 | -0.92 | 0.3601 |
| race | White, Non-Hispanic |  | 0 | . | . | . |
| status | Sexual Minority |  | -0.1510 | 0.1518 | -0.99 | 0.3198 |
| status | Straight |  | 0 | . | . | . |
| age | 18-44y |  | -3.1809 | 0.1143 | -27.83 | <.0001 |
| age | 45-64y |  | -1.0557 | 0.0419 | -25.22 | <.0001 |
| age | 65+y |  | 0 | . | . | . |
| race*age | Black, Non-Hispanic | 18-44y | 0.8811 | 0.2590 | 3.40 | 0.0007 |
| race*age | Black, Non-Hispanic | 45-64y | 0.3769 | 0.1801 | 2.09 | 0.0363 |
| race*age | Black, Non-Hispanic | 65+y | 0 | . | . | . |
| race*age | Other | 18-44y | 0.9758 | 0.2280 | 4.28 | <.0001 |
| race*age | Other | 45-64y | 0.2395 | 0.1572 | 1.52 | 0.1276 |
| race*age | Other | 65+y | 0 | . | . | . |
| race*age | White, Non-Hispanic | 18-44y | 0 | . | . | . |
| race*age | White, Non-Hispanic | 45-64y | 0 | . | . | . |
| race*age | White, Non-Hispanic | 65+y | 0 | . | . | . |
| status*age | Sexual Minority | 18-44y | 0.3212 | 0.3527 | 0.91 | 0.3624 |
| status*age | Sexual Minority | 45-64y | 0.0202 | 0.2099 | 0.10 | 0.9235 |
| status*age | Sexual Minority | 65+y | 0 | . | . | . |
| status*age | Straight | 18-44y | 0 | . | . | . |
| status*age | Straight | 45-64y | 0 | . | . | . |
| status*age | Straight | 65+y | 0 | . | . | . |

**S3.2. Myocardial infarction in females**

| Analysis of Maximum Likelihood Estimates | | | | | | |
| --- | --- | --- | --- | --- | --- | --- |
| Parameter |  |  | Estimate | Standard Error | t Value | Pr > \|t\| |
| Intercept |  |  | -2.6078 | 0.0307 | -85.04 | <.0001 |
| race | Black, Non-Hispanic |  | 0.2619 | 0.1024 | 2.56 | 0.0105 |
| race | Other |  | 0.0818 | 0.1224 | 0.67 | 0.5042 |
| race | White, Non-Hispanic |  | 0 | . | . | . |
| status | Sexual Minority |  | 0.5200 | 0.2755 | 1.89 | 0.0590 |
| status | Straight |  | 0 | . | . | . |
| age | 18-44y |  | -2.5141 | 0.1508 | -16.67 | <.0001 |
| age | 45-64y |  | -0.8314 | 0.0574 | -14.48 | <.0001 |
| age | 65+y |  | 0 | . | . | . |
| race*age | Black, Non-Hispanic | 18-44y | -0.2289 | 0.2991 | -0.77 | 0.4441 |
| race*age | Black, Non-Hispanic | 45-64y | -0.0471 | 0.1520 | -0.31 | 0.7566 |
| race*age | Black, Non-Hispanic | 65+y | 0 | . | . | . |
| race*age | Other | 18-44y | 0.3431 | 0.2815 | 1.22 | 0.2229 |
| race*age | Other | 45-64y | 0.0238 | 0.1783 | 0.13 | 0.8937 |
| race*age | Other | 65+y | 0 | . | . | . |
| race*age | White, Non-Hispanic | 18-44y | 0 | . | . | . |
| race*age | White, Non-Hispanic | 45-64y | 0 | . | . | . |
| race*age | White, Non-Hispanic | 65+y | 0 | . | . | . |
| status*age | Sexual Minority | 18-44y | -0.1599 | 0.3910 | -0.41 | 0.6826 |
| status*age | Sexual Minority | 45-64y | 0.0401 | 0.3544 | 0.11 | 0.9100 |
| status*age | Sexual Minority | 65+y | 0 | . | . | . |
| status*age | Straight | 18-44y | 0 | . | . | . |
| status*age | Straight | 45-64y | 0 | . | . | . |
| status*age | Straight | 65+y | 0 | . | . | . |

**S3.3. Stroke in males**

| Analysis of Maximum Likelihood Estimates | | | | | | |
| --- | --- | --- | --- | --- | --- | --- |
| Parameter |  |  | Estimate | Standard Error | t Value | Pr > \|t\| |
| Intercept |  |  | -2.4770 | 0.0321 | -77.09 | <.0001 |
| race | Black, Non-Hispanic |  | 0.4563 | 0.1233 | 3.70 | 0.0002 |
| race | Other |  | -0.0190 | 0.1208 | -0.16 | 0.8753 |
| race | White, Non-Hispanic |  | 0 | . | . | . |
| status | Sexual Minority |  | 0.2388 | 0.1867 | 1.28 | 0.2008 |
| status | Straight |  | 0 | . | . | . |
| age | 18-44y |  | -2.5877 | 0.1309 | -19.76 | <.0001 |
| age | 45-64y |  | -0.8525 | 0.0563 | -15.15 | <.0001 |
| age | 65+y |  | 0 | . | . | . |
| race*age | Black, Non-Hispanic | 18-44y | 0.3996 | 0.3613 | 1.11 | 0.2687 |
| race*age | Black, Non-Hispanic | 45-64y | 0.1272 | 0.1684 | 0.76 | 0.4499 |
| race*age | Black, Non-Hispanic | 65+y | 0 | . | . | . |
| race*age | Other | 18-44y | 0.3608 | 0.2596 | 1.39 | 0.1645 |
| race*age | Other | 45-64y | 0.2585 | 0.1750 | 1.48 | 0.1396 |
| race*age | Other | 65+y | 0 | . | . | . |
| race*age | White, Non-Hispanic | 18-44y | 0 | . | . | . |
| race*age | White, Non-Hispanic | 45-64y | 0 | . | . | . |
| race*age | White, Non-Hispanic | 65+y | 0 | . | . | . |
| status*age | Sexual Minority | 18-44y | 0.0140 | 0.3953 | 0.04 | 0.9718 |
| status*age | Sexual Minority | 45-64y | 0.0163 | 0.3097 | 0.05 | 0.9580 |
| status*age | Sexual Minority | 65+y | 0 | . | . | . |
| status*age | Straight | 18-44y | 0 | . | . | . |
| status*age | Straight | 45-64y | 0 | . | . | . |
| status*age | Straight | 65+y | 0 | . | . | . |

**S3.4. Stroke in females**

| Analysis of Maximum Likelihood Estimates | | | | | | |
| --- | --- | --- | --- | --- | --- | --- |
| Parameter |  |  | Estimate | Standard Error | t Value | Pr > \|t\| |
| Intercept |  |  | -2.5804 | 0.0296 | -87.17 | <.0001 |
| race | Black, Non-Hispanic |  | 0.4421 | 0.0925 | 4.78 | <.0001 |
| race | Other |  | 0.1388 | 0.2190 | 0.63 | 0.5261 |
| race | White, Non-Hispanic |  | 0 | . | . | . |
| status | Sexual Minority |  | 0.1456 | 0.3082 | 0.47 | 0.6367 |
| status | Straight |  | 0 | . | . | . |
| age | 18-44y |  | -2.2048 | 0.1012 | -21.79 | <.0001 |
| age | 45-64y |  | -0.7286 | 0.0560 | -13.00 | <.0001 |
| age | 65+y |  | 0 | . | . | . |
| race*age | Black, Non-Hispanic | 18-44y | 0.1048 | 0.2152 | 0.49 | 0.6261 |
| race*age | Black, Non-Hispanic | 45-64y | 0.1888 | 0.1337 | 1.41 | 0.1578 |
| race*age | Black, Non-Hispanic | 65+y | 0 | . | . | . |
| race*age | Other | 18-44y | -0.0756 | 0.2961 | -0.26 | 0.7984 |
| race*age | Other | 45-64y | -0.1493 | 0.2527 | -0.59 | 0.5548 |
| race*age | Other | 65+y | 0 | . | . | . |
| race*age | White, Non-Hispanic | 18-44y | 0 | . | . | . |
| race*age | White, Non-Hispanic | 45-64y | 0 | . | . | . |
| race*age | White, Non-Hispanic | 65+y | 0 | . | . | . |
| status*age | Sexual Minority | 18-44y | 0.2931 | 0.3775 | 0.78 | 0.4375 |
| status*age | Sexual Minority | 45-64y | 0.1589 | 0.3642 | 0.44 | 0.6626 |
| status*age | Sexual Minority | 65+y | 0 | . | . | . |
| status*age | Straight | 18-44y | 0 | . | . | . |
| status*age | Straight | 45-64y | 0 | . | . | . |
| status*age | Straight | 65+y | 0 | . | . | . |
